# Impaired liver function in *Xenopus tropicalis* exposed to benzo[a]pyrene: transcriptomic and metabolic evidence

**DOI:** 10.1186/1471-2164-15-666

**Published:** 2014-08-08

**Authors:** Christophe Regnault, Isabelle AM Worms, Christine Oger-Desfeux, Christelle MelodeLima, Sylvie Veyrenc, Marie-Laure Bayle, Bruno Combourieu, Aurélie Bonin, Julien Renaud, Muriel Raveton, Stéphane Reynaud

**Affiliations:** University Grenoble Alpes, LECA, F-38000 Grenoble, France; CNRS, LECA, F-38000 Grenoble, France; University Grenoble Alpes, BEeZy, F-38000 Grenoble, France; Université de Genève, Institute F.A. Forel, Versoix, Suisse; Pôle Rhône Alpes de Bioinformatique, Université Claude Bernard Lyon 1, Villeurbanne, France; Plateforme de recherche en toxicologie environnementale et écotoxicologie de Rovaltain, Valence, France; Laboratoire d’Ecologie Alpine (LECA), UMR CNRS-Université de Grenoble 5553, Domaine Universitaire de Saint-Martin d’Hères, 2233, rue de la piscine Bât D Biologie, BP 53, 38041 Grenoble Cedex 9, France

**Keywords:** RNA sequencing, *Xenopus*, Liver, Metabolic disorders, Insulin resistance-like syndrome, Benzo[a]pyrene

## Abstract

**Background:**

Despite numerous studies suggesting that amphibians are highly sensitive to cumulative anthropogenic stresses, the role pollutants play in the decline of amphibian populations remains unclear. Amongst the most common aquatic contaminants, polycyclic aromatic hydrocarbons (PAHs) have been shown to induce several adverse effects on amphibian species in the larval stages. Conversely, adults exposed to high concentrations of the ubiquitous PAH, benzo[a]pyrene (BaP), tolerate the compound thanks to their highly efficient hepatic detoxification mechanisms. Due to this apparent lack of toxic effect on adults, no studies have examined in depth the potential toxicological impact of PAH on the physiology of adult amphibian livers. This study sheds light on the hepatic responses of *Xenopus tropicalis* when exposed to high environmentally relevant concentrations of BaP, by combining a high throughput transcriptomic approach (mRNA deep sequencing) and a characterization of cellular and physiological modifications to the amphibian liver.

**Results:**

Transcriptomic changes observed in BaP-exposed *Xenopus* were further characterized using a time-dependent enrichment analysis, which revealed the pollutant-dependent gene regulation of important biochemical pathways, such as cholesterol biosynthesis, insulin signaling, adipocytokines signaling, glycolysis/gluconeogenesis and MAPK signaling. These results were substantiated at the physiological level with the detection of a pronounced metabolic disorder resulting in a possible insulin resistance-like syndrome phenotype. Hepatotoxicity induced by lipid and cholesterol metabolism impairments was clearly identified in BaP-exposed individuals.

**Conclusions:**

Our data suggested that BaP may disrupt overall liver physiology, and carbohydrate and cholesterol metabolism in particular, even after short-term exposure. These results are further discussed in terms of how this deregulation of liver physiology can lead to general metabolic impairment in amphibians chronically exposed to contaminants, thereby illustrating the role xenobiotics might play in the global decline in amphibian populations.

**Electronic supplementary material:**

The online version of this article (doi:10.1186/1471-2164-15-666) contains supplementary material, which is available to authorized users.

## Background

Dramatic declines in amphibian populations in wetlands have been described worldwide since the 1980s [1], with current extinction rates reaching levels that are 211 times higher than background levels [2]. Despite lengthy debate about causative agents responsible for this species decline, theoretical and empirical research has confirmed the complex cross-effects of anthropogenic activities on the survival of amphibians through habitat loss, the introduction of exotic species, increased UV radiation, water acidification, emerging infectious diseases and multi-pollution [3–5]. Some field studies using landscape-scale data have suggested that chemical contaminants may correlate with population declines in several amphibian species [6–9]. Therefore, chemical contaminants were proposed as one of the key contributing factors altering amphibian population fitness [10]. However, the exact mechanisms of population responses to chemical exposure are still poorly understood, leading several authors to consider pollution as a cause of secondary importance [11].

Numerous toxicological studies have reported diverse physiological effects in individuals exposed to pollutants, such as increased mortality, decreased growth rates, malformations, endocrine disruption and immunosuppression [3–5]. Most of these studies solely investigated larval exposure and focused on pesticides as contaminants [5]. However, freshwater ecosystems are polluted with complex mixtures including emerging and well-known pollutants, like heavy metals, polychlorobiphenyls, personal care products, drugs and polycyclic aromatic hydrocarbons (PAHs), as well as their transformation products. In the case of PAHs, several studies have suggested that amphibians exhibit a variety of physiological disorders when exposed to environmentally relevant concentrations of PAHs (from 25 ng.L^-1^ to 30 μg.L^-1^) at the larval stage [12–15]. However few studies have focused on adult amphibian responses [16–18] and even less on the all-important females on which the resources found in the eggs are entirely dependent [19]. The PAH, benzo[a]pyrene (BaP) is classified as a priority pollutant, based on its known carcinogenicity [20, 21]. The mechanisms causing BaP toxicity are linked to its bioactivation through a series of enzymes specifically induced for the metabolism and excretion of BaP, as the genes encoding for enzymes are closely- regulated by AhR and Nrf2 transcription factors [22–24]. One deleterious effect of this active metabolism is the generation of genotoxic (capable of forming DNA adducts) and non-genotoxic metabolites, some of which are capable of inducing oxidative stress [25]. Since the main BaP and other xenobiotic biotransformation reactions take place in the liver, this is also the preferential location for collateral toxic events [26]. It would appear that the bioaccumulation of BaP is very pronounced in amphibian species [27], but adult females exposed to relatively high doses (10 μg.L^-1^) of BaP presented good levels of tolerance to this contaminant due to efficient hepatic detoxification [16]. To date, in the absence of any apparently toxic effects linked to the specific aforementioned BaP regulation, no study has assessed the possible hidden harm to female amphibian liver physiology caused by PAHs. Today, this knowledge gap can be bridged using high throughput sequencing approaches, such as mRNA sequencing (mRNAseq), recovering information on gene expression over the whole transcriptome from a single experiment [28–31]. This technique has recently been shown to be more sensitive, reliable and informative than microarrays for the *in vitro* evaluation of BaP toxicity [32]. In the case of *X. tropicalis*, the interpretation of large numbers of sequences generated by mRNAseq takes advantage of the recently released complete genome [33], and benefits from a comprehensive repository from the standard model *X. tropicalis,* giving access to all levels of sequence data sets, including transcriptomic data [34]. *X. tropicalis* is easy to maintain, has a short life cycle and is an appropriate model for the analysis of the sublethal effects of toxicants in amphibians [35, 36]. *X. tropicalis* would therefore appear to be an excellent amphibian model for in-depth studies on the more hidden effects of chemical contaminants, particularly BaP, on the female liver at different biological levels.

The objective of this study was to reveal *in vivo* hepatic responses in *X. tropicalis* females exposed to an environmentally relevant concentration of BaP (10 μg.L^-1^) at the transcriptomic, cellular and physiological levels. Due to the dynamic nature of cellular responses, a kinetic approach was applied over a 24-hour exposure period by sequencing mRNA at 6, 12, 18 and 24 hours post-exposure. Transcriptomic data collected from exposed and non-exposed animals were compared in order to identify hepatic metabolic pathways significantly affected by BaP treatment. The metabolic pathways which were found to be enriched at transcriptomic level were studied in detail as regards their chronological activation by BaP, and were further associated to cytological and physiological liver phenotype changes over the same time period.

## Results

### BaP uptake and metabolism

**Figure 1:**
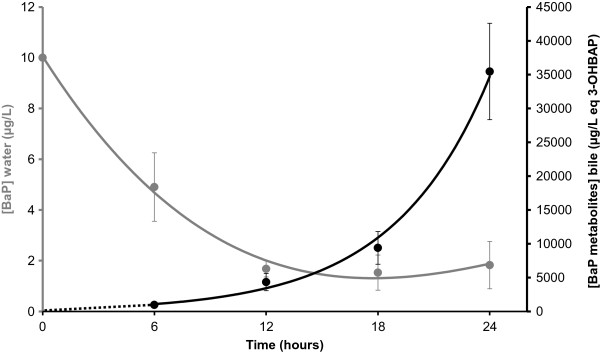
**Kinetics of benzo[a]pyrene (BaP) concentrations in water (grey curve) and metabolite concentrations in bile (black curve) as a function of exposure time during** ***Xenopus tropicalis*** **exposure.** Values are mean ± SE for 3 replicates.

BaP clearance in the water was used as an indicator of the uptake kinetics in *Xenopus* (Figure 1). The observed decrease in BaP concentrations in the water indicated rapid trans-tegumental uptake of the pollutant in the organism with a mean of 0.88 ± 1.14 μg per hour in the first 6 hours. From hour 6 to 12, the uptake rate dropped to 0.6 μg per hour. From hour 12 to the end of exposure, no further decrease in BaP concentration was observed and a residual concentration of approximately 1.6 μg.L^-1^ was measured, corresponding to 16% of the initial BaP concentration (Figure 1, grey dots).

Hepatic BaP metabolism was evaluated by measuring total BaP metabolites content in the gall bladder (Figure 1, black dots). Benzo[a]pyrene metabolite concentrations in the gall bladder increased concomitantly with the depletion of BaP in the water. The liver metabolism rate drastically increased after 12 hours of exposure, which corresponds to the point of maximum BaP depletion from the water. The highest liver metabolism rate was observed from hour 18 to 24 post-exposure, with 65 ng of BaP metabolized per hour.

### Sequencing and mapping of cDNAs

By sequencing 8 cDNA libraries from non-exposed control and exposed *Xenopus* to 10 μg.L^-1^ BaP at hours 6, 12, 18 and 24, a mean of 35.1 million reads per library were sequenced. Approximately 80% of these reads were successfully mapped to the *X. tropicalis* genome and a mean of 19.51 million of reads were mapped onto known genes at a single genomic location with no ambiguity (Additional file 1: Figure S1A). A total of 13,551 genes were represented by more than 20 reads across all libraries (Additional file 1: Figure S1B and Additional file 2: Table S1). An analysis of transcription levels from hour 6 to 24 identified 308 genes differentially expressed (FDR < 0.05) with a transcript ratio (TR) > 1.5-fold in either direction at at least one time point during exposure (Additional file 1: Figure S1B and Additional file 3: Table S2). Excepted for hour 12 of exposure when the majority of genes were over-transcribed, the distribution of transcription ratios was well balanced between under- and over-transcription with most TRs being within a 5-fold variation in either direction. Interestingly, only four genes were consistently found to be differentially transcribed at the four time points considered, and an average of 5 genes were found consistently differentially transcribed at two or three time points with a maximum of 20 genes shared between hour 6 and 12 (Additional file 1: Figure S1C). Cross-validation of TRs from mRNA sequencing with real-time quantitative RT-qPCR at each point of exposure revealed a positive correlation between TRs obtained from these two techniques in over 10 genes studied (r = 0.89 and p <0.001) (Additional file 4: Figure S2).

### Functional analysis of BaP regulated genes and their dynamics during exposure

Among the 308 genes differently transcribed at at least one time point during BaP exposure, 62 of them encoded proteins with unknown functions (Additional file 3: Table S2). Approximately 20% encoded proteins involved in “signaling/transport” and in “cell death/proliferation processes” (Additional file 5: Figure S3). Around 29% were assigned to cell metabolism with 28, 16 and 26 genes involved in the metabolism of “lipids”, “carbohydrates” and “proteins”, respectively. Thirty-four, 18 and 10 genes encoded proteins involved in “cell structure”, “immunity” and “DNA interaction and repair”, respectively. Interestingly, only 12 genes encoded proteins involved in detoxification processes (Additional file 5: Figure S3). In addition, the general transcription pattern clearly indicated over-transcription at 12 hours post-exposure regardless of the gene function considered (Additional file 5: Figure S3).

However, a manual annotation only highlights the general functions associated with gene transcription, and does not identify the biochemical pathways in which proteins may be involved. An annotation enrichment analysis was therefore performed in order to determine whether the genes associated with KEGG pathways (Kyoto Encyclopedia of Genes and Genomes) were significantly over-represented in differentially transcribed genes. An initial assignment of the 308 *Xenopus* genes using their Ensembl identifiers yielded less than 6.7% of genes recognized by the functional annotation tool DAVID (Database for Annotation, Visualisation and Integrated Discovery). To compensate for the small number of directly attributed terms, the *Xenopus* genes detected in our study (13,357) were mapped against their closest human orthologs (Additional file 3: Table S2). After manual verification of each gene to check for similarity in terms of function and description, 78.4% of *Xenopus* genes had human orthologs (10,483) of which 10,322 (98.4%) matched with annotated DAVID gene objects. Among the genes found to be significantly differentially transcribed, 207 of the 246 with known descriptions (84%) matched with annotated DAVID gene objects. Annotation enrichment analyses performed on over-transcribed genes indicated a link between BaP treatment and “steroid biosynthesis”, “insulin signaling pathway”, “adipocytokines signaling pathway”, “glycolysis/gluconeogenesis” and “MAPK signaling pathways” (Table 1). Interestingly, annotation enrichment analyses performed on under-transcribed-genes again indicated a link between BaP treatment and “steroid biosynthesis” pathways as well as “tight junction” pathways (Table 1).

**Table 1:** **Pathway enrichment study based on genes differentially transcribed between control and BaP-exposed animals**

	Kegg pathway	Number of genes	Fold enrichment	p value ^*^
**UP**	Steroid biosynthesis (cholesterol biosynthesis)	7	23.8	1.6E-07
	Insulin signaling pathway	7	3.1	2.3E-02
	Adipocytokine signaling pathway	5	5	1.6E-02
	MAPK signaling pathway	9	2.5	2.4E-02
	Glycolysis / Gluconeogenesis	4	4.5	5E-02
**DOWN**	Steroid biosynthesis (cholesterol biosynthesis)	4	27.8	3.3E-04
	Tight junction	6	5.3	4.6E-03

The 308 genes showing a significant differential transcription at least one time point were used for annotation enrichment using the DAVID (Database for Annotation, Visualisation and Integrated Discovery) functional annotation tool. ^*^modified Fisher’s Exact test (p < 0.05).

### Effects of BaP on the steroid biosynthesis pathway and lipid metabolism

**Figure 2:**
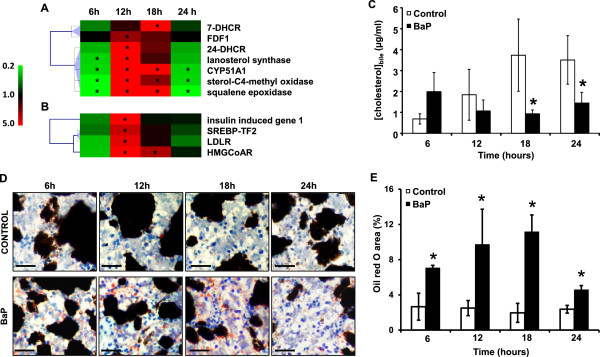
**Lipid metabolism disorder induced by BaP. A**. Hierarchical clustering of cholesterol biosynthesis genes differentially transcribed compared to control. The color scale indicates transcription ratios relative to the control. Gene names or annotations are indicated. *7-DHCR*, 7-dehydrocholesterol reductase; *FDF1*, farnesyl-diphosphate farnesyltransferase 1; *24-DHCR*, 24-dehydrocholesterol reductase; *CYP51A1*, cytochrome P450-51A1. Stars indicate significant transcription variations (>1.5-fold in either direction and adjusted p < 0.05). **B**. Hierarchical clustering of genes involved in cholesterol biosynthesis regulation and depletion from the blood differentially transcribed compared to control. The color scale indicates transcription ratios relative to the control. Gene names or annotations are indicated. *SREBP-TF2*, sterol regulatory element binding transcription factor 2; *LDLR*, low density lipoprotein receptor, *HMGCoAR*, 3-hydroxy-3-methylglutaryl-CoA reductase. Stars indicate significant transcription variations (>1.5-fold in either direction and adjusted p < 0.05). **C**. Kinetics of cholesterol concentration in bile. Data represent mean ± SE values for 3 replicates. Statistical analysis was performed using the Mann–Whitney test, asterisks indicate a significant difference from the control: *, p < 0.05. **D**. Oil red staining for total lipid content measures in the livers of control and BaP-exposed animals. Lipid content was indicated by red staining. Bars = 25 μm. **E**. Percentage of oil red area in the livers of control and BaP-exposed animals. Data are mean ± SE values for 24 sections replicates. Statistical analysis was performed using the Mann–Whitney test (n = 3), and asterisks indicate a significant difference from the control: *, p < 0.05.

The enrichment of KEGG “steroid biosynthesis” pathway concerned genes involved in cholesterol biosynthesis using acetyl-coA as a precursor [*farnesyl-diphosphate farnesyltransferase* 1 (FDF1), *squalene epoxidase, lanosterol synthase*, *cytochrome P450 51A1* (CYP51A1), *24-dehydrocholesterol reductase* (24-DHCR) and *7-dehydrocholesterol reductase* (7-DHCR)]. The dichotomic link between BaP treatment and cholesterol biosynthesis is clearly characterized by two chronological phases of the transcriptional response (Figure 2A). Genes involved in this pathway appeared over-transcribed at 12 and 18 hours post-exposure and under-transcribed at the first (6 hours) and last (24 hours) time points considered in the present study. Interestingly, this gene transcription time pattern was similarly detected for genes involved in cholesterol biosynthesis regulation (*insulin-induced gene 1, SRE-binding transcription factor2 and HMG CoA-reductase*) as well as in cholesterol depletion from the blood (LDL receptor) (Figure 2B).

In the control animals, gall bladder cholesterol concentrations increased from 0.69 μg.mL^-1^ to 3.73 μg.mL^-1^ during the first 18 hours and slightly decreased thereafter, reaching 3.50 μg.mL^-1^ at 24 hours. Bile cholesterol concentrations in the BaP-exposed animals were 4 to 2.5-fold significantly lower than the controls at 18 and 24 hours post-exposure, respectively (Figure 2C).

Histological data revealed that BaP induced an important accumulation of lipid droplets in the liver *i.e.* steatosis (Figure 2D). In BaP-treated animals, a significant increase in hepatocyte lipid contents was observed from 6 to 18 hours post-exposure followed by a significant decrease at 24 hours. At hours 12 and 18 of exposure, hepatocyte lipid content was about 6-fold higher in BaP-treated animals compared to the controls (Figure 2E).

### Effects of BaP on glucose metabolism

**Figure 3:**
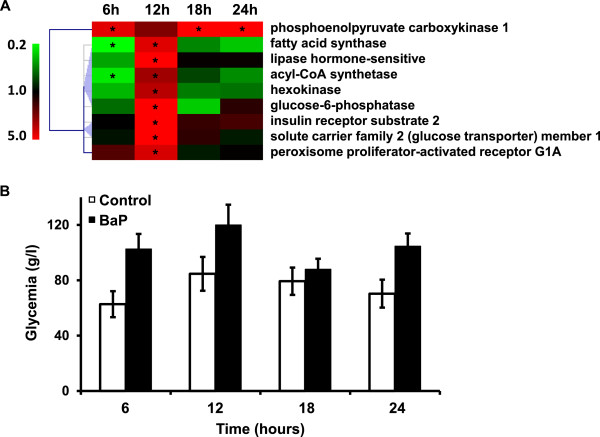
**Carbohydrate metabolism disturbances induced by BaP. A**. Hierarchical clustering of genes found to be differentially transcribed compared to the control and involved in insulin, adipocytokine and gluconeogenesis pathways. Color scale indicates transcription ratios relative to the control. Gene names or annotations are indicated. Stars indicate significant transcription variations (>1.5-fold in either direction and adjusted p < 0.05). **B**. Glycemia kinetics in control and in BaP-exposed *X. tropicalis*. Data are expressed as mean ± SE of 3 replicates.

More than 50% of the genes linked to “insulin signaling”, “adipocytokines signaling” and “glycolysis/gluconeogenesis” pathway enrichment were shared across these three pathways, suggesting that BaP strongly affects glucose metabolism. Interestingly, the genes involved in glycolysis/gluconeogenesis were over-transcribed, especially at 12 hours post-exposure, *phosphoenolpyruvate carboxykinase 1* excepted, which was significantly over-transcribed at 6, 18 and 24 hours post exposure (Figure 3A). Three genes involved in fatty acid and lipid metabolism and closely linked to carbohydrate metabolism were found to be over-transcribed at 12 hours post-exposure (*fatty acid synthase, lipase hormone-sensitive* and *acyl-CoA synthetase*) and two of them (*fatty acid* and *acyl-CoA synthetases*) were found to be under-transcribed at 6 hours post-exposure (Figure 3A).

In addition, the monitoring of glucose concentrations in blood indicated a marked hyperglycemia induced by BaP at 6, 12 and 18 hours post-exposure (Figure 3B).

### Effects of BaP on the MAPK signaling pathway, hepatocyte apoptosis

**Figure 4:**
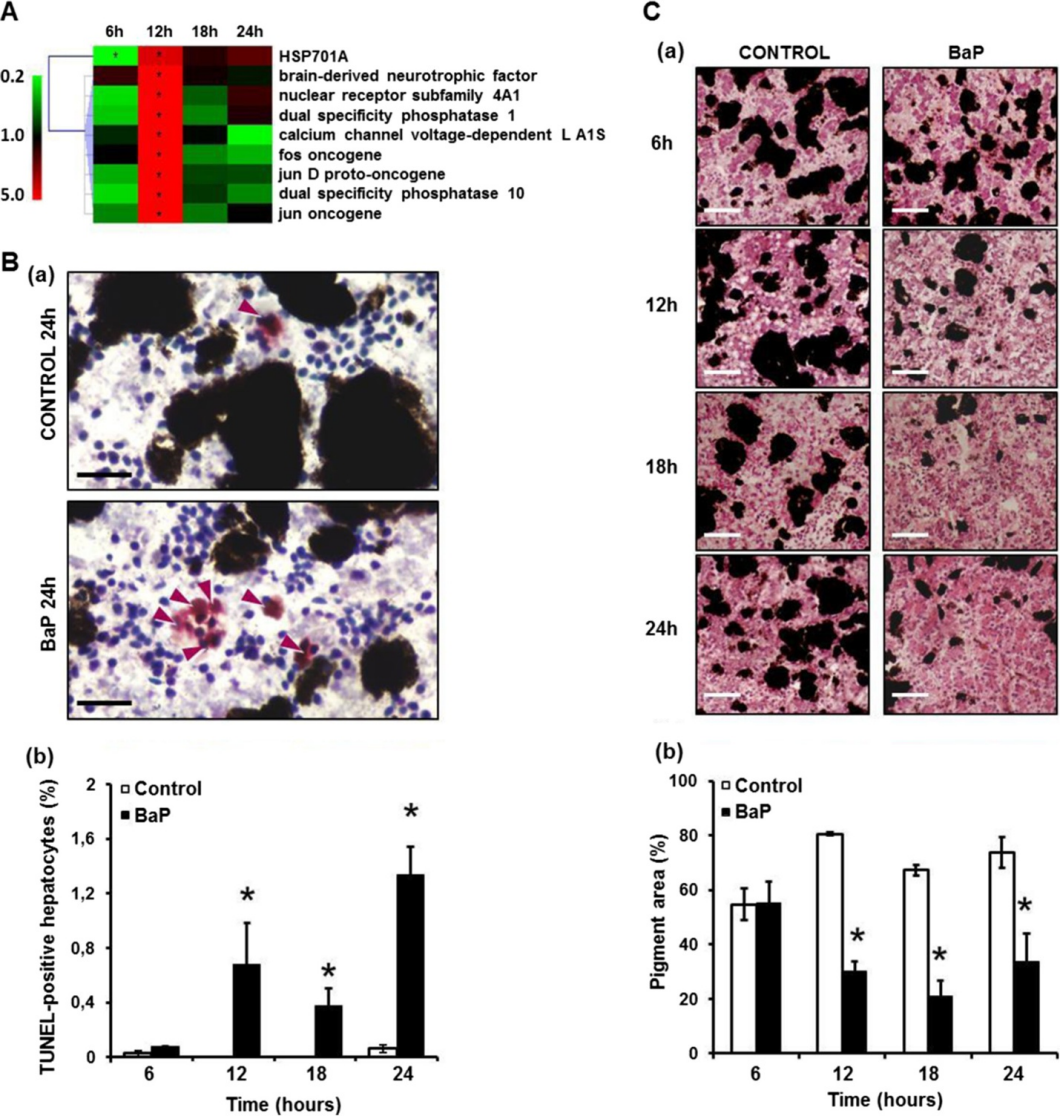
**Hepatotoxicity induced by BaP. A**. Hierarchical clustering of MAPK genes found differentially transcribed compared to the control. Color scale indicates transcription ratios relative to the control. Gene names or annotations are indicated. *HSP701A*, *heat shock protein 701A*. Stars indicate significant transcription variations (>1.5-fold in either direction and adjusted p < 0.05). **B**. (a) TUNEL staining detecting apoptosis-induced DNA damage in hepatocytes in control and in BaP-exposed animals at 24 hours. Arrows indicate apoptotic hepatocytes. Bars = 50 μm. (b) Percentage of TUNEL-positive nuclei in the livers of control and BaP-exposed animals according to the exposure time points. Data are mean ± SE values for 21 sections replicates. Statistical analysis was performed using the Mann–Whitney test, and asterisks indicate a significant difference from the control: *, p < 0.05. **C**. (a) Hematoxylin-eosine-safran (HES) staining of liver sections from control and *X. tropicalis* exposed to BaP. Bars = 100 μm. (b) Percentage of pigment area in liver sections of control and BaP exposed animals. Data are mean ± SE values for 21 sections replicates. Statistical analysis was performed using the Mann–Whitney test (n = 3), and asterisks indicate a significant difference from the control: *, p < 0.05.

Genes involved in the “MAPK signaling” pathway were found to be over-transcribed at 12 hours post-exposure. Only the *HPS701A* gene was also found to be significantly under-transcribed at 6 hours post-exposure (Figure 4A). Since MAPKs are typically associated with cell apoptosis and proliferation, a clustering analysis was carried out on all the genes involved in these processes. The vast majority of genes involved in the apoptosis pathway (21 out of 23) appeared to be over-transcribed at at least one time point, with 91% over-expressed at 12 hours post-exposure (Additional file 6: Figure S4). For genes involved in proliferation processes, the expression pattern was more complex. About 50% of these were over-transcribed at 12 hours post-exposure, whereas 36% were found to be under-transcribed at 6 and 24 hours post-exposure (Additional file 6: Figure S4).

The hepatocyte apoptosis was significantly higher in BaP-exposed *Xenopus* than in the controls at hours 12, 18 and 24 post-exposure. Apoptotic hepatocytes were absent or relatively rare in the controls, but their number significantly increased by 20-fold in exposed individuals at 24 hours post-exposure (Figure 4B).

Liver pigment content is highly variable in amphibians, and its decrease is associated with hepatic stress and hepatocyte apoptosis. *X. tropicalis* liver is highly pigmented with a maximal pigment area covering 55 to 80% of the tissue sections, as observed in the controls. In exposed *Xenopus*, BaP induced a marked decrease in liver pigment content from hours 12 to 24 post-exposure. Pigment areas were found to have decreased 3-fold at 18 hours post-exposure (Figure 4C).

### Effects of BaP on cell-cell interactions

The “tight junction” pathway appeared to be highly down-regulated at all time points, except at 12 hours with the under-transcription of genes involved in interactions between claudins and actin (*myosin heavy chain 7 putative, cold shock domain protein A, myosin heavy chain 4, myosin heavy chain 3, actinin alpha 2*) and in the transport of claudins to cell membranes (*Rab3B*). Interestingly, this phenomenon was concomitant with an under-transcription of *E-cadherin*, a key protein involved in adherent junctions (Additional file 7: Figure S5A). Histological examinations of samples stained with hematoxylin and eosin revealed that the hepatic tissue organization appeared to be destructured in BaP-treated animals. Under magnification, the liver tissue in exposed *Xenopus* displayed hepatocytes with fewer cell contacts and irregular shapes (Additional file 7: Figure S5B).

## Discussion

Despite evidence suggesting that amphibians are very sensitive to pollutants [5], their role in the decline of amphibian populations remains unclear [11]. In this study, we used the carcinogenic pollutant BaP to attempt to uncover any potential hidden toxic effects it may have on the liver tissue of the amphibian model *X. tropicalis* under sub-lethal exposure. The concentration of BaP used in this study has been calculated based on previous studies on green frogs [16] and the environmental concentrations of BaP likely to occur in highly-polluted sites where frogs are found [14, 27, 37]. Here, we emphasize the modulation of the transcriptome and liver cellular/physiological phenotype changes over a 24-hour exposure time period. This short-term exposure has been selected based on the results of previous studies on green frogs [16] and in order to mimic acute exposure.

Recent *in vitro* studies have clearly illustrated the dynamic regulation occurring at the transcriptomic level during BaP exposure and the sequence of events leading to BaP toxicity [32]. Similarly, our results highlighted remarkable variations in hepatic gene transcription during pollutant exposure, with major changes occurring at 12 hours post-exposure.

The rapid uptake of BaP from contaminated water and the early excretion of BaP metabolites into the bile observed in the *Xenopus* used in this study, testify to the active involvement of cohorts of specific oxidases, transferases and transport proteins necessary for BaP metabolism. However, none of the 308 genes differentially transcribed upon BaP exposure belonged to the “xenobiotic metabolism” pathway, such as *ahr*, *cyp1A1*, *gstm1* or *ugt1a1*. Moreover, genes associated with biotransformation pathways are known to be involved in the metabolism of endogenous compounds rather than xenobiotics. The induction of BaP metabolism enzymes has been previously shown to occur rapidly after pollutant exposure. In the hepatic cellular model HepG2 for instance, a rapid increase in the expression of genes involved in “Dioxin, xenobiotic metabolism” was observed to drop over the first 9 hours after exposure to 751 μg.L^-1^ of BaP [32]. In fish exposed to a high concentration of BaP (5 mg.L^-1^), one of the main oxidases involved in phase I of the BaP metabolism process, encoded by the *CYP1A* gene, was over-transcribed immediately after BaP exposure with a high TR of ~ 46, decreasing to 10 6 hours post-exposure [38]. The lack of “xenobiotic metabolism” pathway genes in our data might be due to the low concentration of BaP applied to *Xenopus*, as well as the relatively long 6-hour period before sampling at the first experimental time point. Detecting immediate BaP metabolism in *X. tropicalis* and early gene regulation by this PAH is therefore unfeasible. Our findings do, however, concur with previous studies on frogs showing rapid fecal elimination of BaP and a relatively good tolerance of this xenobiotic [16]. This further suggested that the classical molecular biomarkers of PAH exposure, such as *CYP1A* induction, may not be sufficient to highlight liver responses under short-term exposure at low concentrations.

It is widely known that the liver plays a key role in lipogenesis, gluconeogenesis and cholesterol metabolism. The associated metabolic pathways are closely regulated by insulin or adipocytokines [39]. In our study, a significant enrichment of insulin signaling, adipocytokine signaling, glycolysis/gluconeogenesis, steroid (cholesterol) biosynthesis, and MAPK signaling pathways was observed; this may relate to late transcriptomic responses to BaP exposure in *in vitro* models [32]. To date, the *in vivo* kinetics of mechanisms regulated by BaP exposure have not been investigated.

The pathways enriched must be considered as a whole and suggest that a possible insulin resistance-like (IR) phenotype occurs rapidly in *X. tropicalis* exposed to BaP. Insulin resistance is characterized by an increase in the enzyme-coding transcripts involved in gluconeogenesis, like *phosphoenolpyruvate carboxykinase* (PEPCK), and by a sustained hyperglycemia [39]. Indeed, we found a marked over-transcription of the *PEPCK* gene as early as 6 hours post-BaP-exposure, in addition to a marked hyperglycemia at hours 6, 12 and 24 post-exposure. These findings were confirmed by the over-transcription in BaP-exposed *Xenopus* of the *glucose-6 phosphatase* gene coding for a major gluconeogenic enzyme [39] and of the glucose transporter GLUT2 [40]. Moreover, the high over-transcription we observed at 6 hours post-exposure for the insulin-like growth factor 1 binding protein (IGFBP-1) repressed by insulin in physiological conditions [41], indicated a marked responsiveness of cell receptors liable to early IR syndrome within a short exposure period. Another important physiological feature is the occurrence of liver steatosis, which is the direct consequence of triglyceride accumulation, observed at the earliest time point considered *i.e.* 6 hours of pollutant exposure. Liver steatosis is a well-known consequence of exposure to xenobiotics as evidenced in fish exposed to mercury or large concentrations of BaP [26, 42, 43]. Moreover, liver steatosis has been shown to occur within a few hours of mammals or amphibians being subjected to chemical or hypoxic stress [44–46]. More interestingly, steatosis was shown to be a symptom of IR [39, 47]. Based on our transcriptomic results, this phenomenon can be directly related to the under-transcription of *sulfotransferase 2B1* (*SULT2B1*) at 6 hours post-BaP-exposure, an enzyme known to protect the liver from steatosis and to inhibit lipogenesis [48].

In our study, the majority of hepatic genes modulated on BaP exposure led to a significant enrichment of steroid (cholesterol) biosynthesis, with a marked under-transcription at 6 and 24 hours, and over-transcription at 12 and 18 hours. Sustained hyperinsulinemia, a characteristic syndrome of IR, can promote the accumulation of liver cholesterol through the activation of the *sterol regulatory element binding transcription factor 2* (*SREBP-TF2*) [47]. Indeed, our results indicated a marked over-transcription of *SREBP-TF2* 12 hours post-exposure leading to the over-transcription of *HMGCoA reductase* and *LDLR* involved in hepatic cholesterol biosynthesis and cholesterol uptake from blood, respectively, at hours 12 and 18 post-exposure. This phenomenon has been previously demonstrated in mammals and frogs [19, 48]. Consequently, the genes involved in cholesterol biosynthesis with acetyl-CoA as a precursor were found to be up-regulated. The induction of cholesterol biosynthesis has been previously suggested as a marker of liver toxicity from lead, but the underlying mechanisms have not been explored [49]. In addition, a previous study on mammals [50] showed that fasting, as implemented in our experimental conditions, induced an increase in cholesterol output in the bile. This phenomenon was observed in the unexposed *Xenopus* from 6 to 18 hours after food was withdrawn. However, in exposed animals, a decrease in biliary cholesterol clearly indicated a disruption in cholesterol export through the gall bladder. Since hyperinsulinemia is known to induce a reduction in free cholesterol efflux into the bile [47], this latter result reinforces our hypothesis that IR occurs and is one of the possible causal effects of the cholesterol metabolism disorder observed after 12 hours of exposure to BaP.

A major consequence of steatosis and cholesterol accumulation in the liver is the induction of hepatic apoptosis/necrosis. Hepatocellular accumulation of lipid droplets predisposes to the overproduction of reactive oxygen species (ROS), whereas cholesterol accumulation induces endoplasmic reticulum (ER) stress, ultimately leading to hepatocyte apoptosis/necrosis and autophagy [39, 47]. In our study, liver steatosis and the induction of the cholesterol biosynthesis and uptake pathways were accompanied by the induction of hepatocyte apoptosis, a loss in liver pigments and an induction of ROS scavenger enzyme-coding gene expression, such as *catalase*. The amphibian livers are characterized by the presence of large quantities of pigments synthesized by melano-macrophagic cells. Pigment content in the liver is highly dynamic and varies according to natural (hibernation) or pathological (infections etc.) conditions. A decrease in liver pigments has been previously associated with metabolic stress, apoptosis and autophagy [51]. Overall, our results indicated that the perturbation of lipid metabolism induced by BaP was responsible for lipotoxic phenomena including ER stress, ROS production and apoptosis. These results were confirmed by a high over-transcription of pro-apoptotic genes (*tumor protein p53 inducible nuclear protein* and *cyclin L2*) [52, 53] and the down-regulation of genes involved in tight and adherent junctions [42]. In the liver, the cohesion of parenchymal hepatocytes and biliary epithelial cells is maintained by apical junctional complexes, such as tight and adherent junctions [54]. The irregular shape and loose contact with neighboring cells that were observed, corroborated by the down-regulation of junction-linked genes, could be attributed to the induction of apoptotic processes [42].

Among the key proteins which regulate apoptosis/proliferation, MAPKs are closely linked to insulin signaling in the liver. Previous studies have described changes in the liver in response to insulin including glycolysis induction and MAPK–ERK signaling pathway leading to cell proliferation [55]. However, the MAPK signaling pathway is classically defined as a key regulator of the cellular balance between apoptosis and proliferation [56]. Our results highlight the transcription of key oncogenes involved in cell proliferation (*fos*) and apoptosis (*jun* D). These results suggest a possible balance between cell proliferation induced by hyperinsulinemia, and apoptosis induced by lipotoxicity and ER stress [39, 47, 55]. However, since we did not observe any hepatocyte proliferation at the histological level, and given the measurement of proliferative cell nuclear antigen (*PCNA*) (data not shown), our results indicate that the pro-apoptotic branch of the MAPK pathway predominated in BaP-treated *Xenopus*.

The hypothesis for this study is that IR-like symptoms will develop rapidly in *Xenopus* exposed to BaP, affecting the metabolism of carbohydrates and fatty acids and thus leading further to liver steatosis with an apoptotic phenotype in hepatocytes. Cholesterol metabolism disruption is known to be associated with IR [47], and our temporal study shows that this occurs posterior to the IR symptoms. As it is the case in mammals [57], cholesterol metabolism in frogs is tightly regulated by estrogens. More specifically, estrogens induce an increase in hepatic cholesterol synthesis and uptake by activating *HMGCoA reductase* and *LDLR* gene transcription, allowing the lipidation of vitellogenin, which is involved in egg maturation [19]. Moreover, estrogen receptor signaling is affected by IGF-1 in a feedback loop [57] and AhR regulation [58], the latter being involved in the disruption of estrogen-induced vitellogenin synthesis in chicken and fish [59, 60]. Steatosis and lipid metabolism disruption has also been associated with other, inter-connected, endocrine axes [61]. We thus hypothesized that the “outbreak of gene expression” observed at 12 hours post-exposure, associated with lipid and cholesterol metabolism, could also be explained by a disruption of the homeostatic mechanisms in other organs, and the result of a multi-signal integration leading to a perceptible subsequent impact on the liver transcriptome. For example, an increase in the transcription of the *UDPGT2A1* gene encoding an enzyme responsible for estrogen conjugation and elimination by the liver was observed [62, 63], somewhat contradicting the observation of over-transcription of the *lipase hormone sensitive* gene encoding an esterase involved in the discharge of cholesterol for further use as a steroid precursor [64].

The underlying mechanisms remain unclear and warrant further investigation. The marked induction of the hepatic cholesterol biosynthesis pathway triggered by BaP may ultimately interfere with the estrogen-regulated lipidation of vitellogenin. In the long term, such mechanisms may impair amphibian reproduction by disrupting estrogen-controlled egg maturation.

## Conclusions

**Figure 5:**
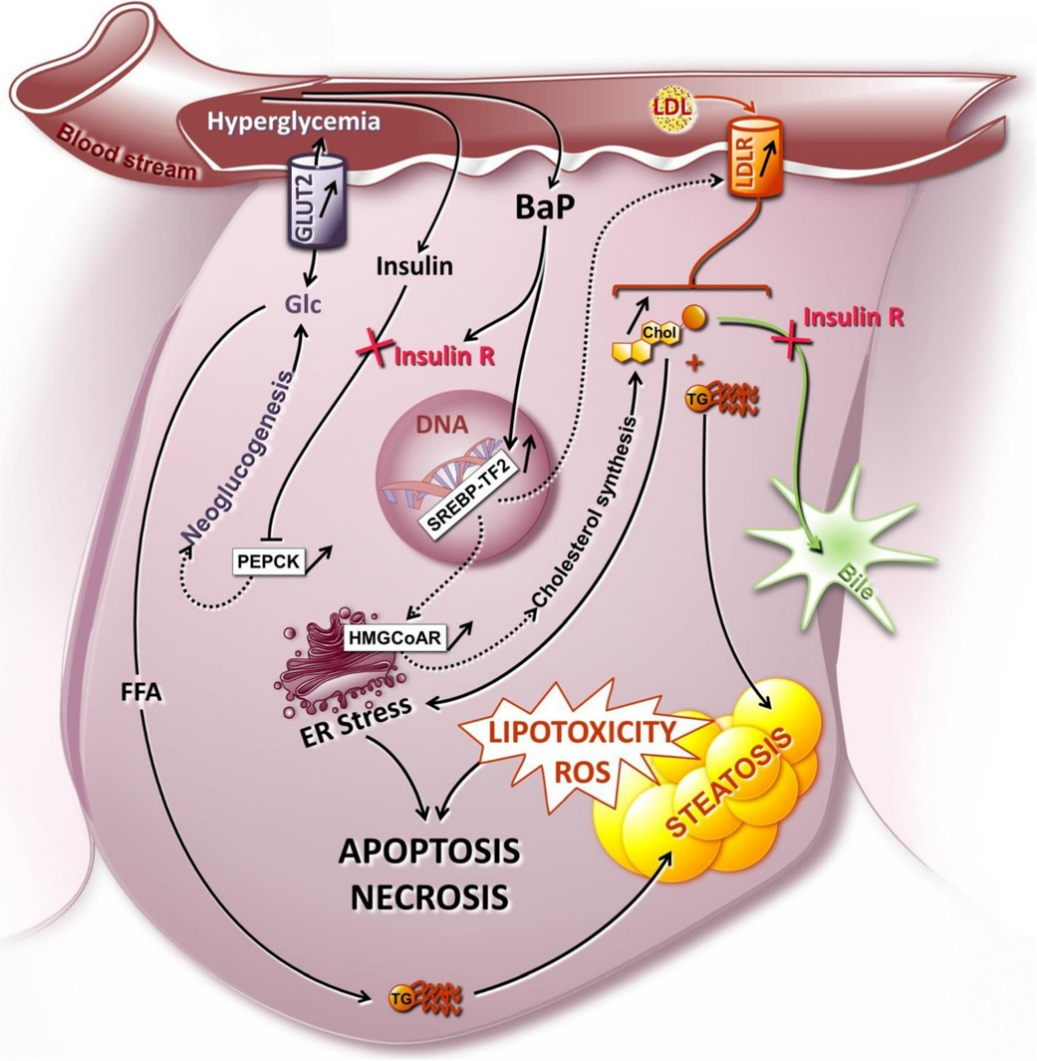
**Cellular pathways potentially involved in** ***X. tropicalis*** **hepatocyte responses to BaP exposure at sub-lethal concentration.** This model, based on our transcriptome and liver phenotype dynamic analyses, suggests that BaP is responsible for the induction of an insulin-resistance-like phenotype in *Xenopus*. Insulin resistance is characterized by the over-transcription of gluconeogenic enzymes genes (*PEPCK*), sustained hyperglycemia, an over-transcription of glucose transporter (*GLUT2*) and severe liver steatosis. Consequently, hyperinsulinemia may lead to the marked induction of cholesterol synthesis pathways and to a decrease in cholesterol export to the bile. The accumulation of lipids and cholesterol in the hepatocytes thus induces both ER stress and lipid toxicity leading to apoptosis or necrosis. *LDL*, Low density lipoprotein; *LDLR*, Low density lipoprotein receptor; *GLUT2*, Glucose transporter 2; Glc, glucose; Chol, cholesterol; *PEPCK*, phosphoenolpyruvate carboxykinase 1; *HMGCoAR*, 3-hydroxy-3-methylglutaryl-CoA reductase; *SREBP-TF2*, sterol regulatory element binding transcription factor 2; ER, endoplasmic reticulum; FFA, Free fatty acid, TG, triglycerides; Insulin R, insulin resistance.

This study provides initial insights into the hidden adverse effects of high environmental concentrations of BaP on anurans, using *X. tropicalis* as a biological model. Although decreased cholesterol in the bile and an increase in steatosis are known consequences of xenobiotic exposure, our kinetic approach and the use of mRNA sequencing revealed for the first time the sequential toxic effect mechanisms induced by PAHs. BaP induced a marked metabolic disorder in the liver highlighted by an insulin resistance-like syndrome phenotype and hepatotoxicity due to impaired lipid metabolism (Figure 5). Although the concentration used in this study is likely to occur in highly-polluted sites where frogs live and reproduce, a 24-hour exposure is more characteristic of acute contamination. Additional studies concerning chronic exposure to commonly-occurring environmental concentrations of BaP are needed to confirm the relevance of our results to *in situ* conditions. However, taken overall, our results strongly suggest that BaP may disrupt carbohydrate and cholesterol metabolism, and more generally liver physiology as a whole, even after short-term exposure. In the long term, such mechanisms may impair amphibian physiology and reproduction by disrupting egg maturation and energy allocation for reproduction.

## Methods

### Animals

*Xenopus* (*X. tropicalis*) females were purchased from the “*Xenopus* national husbandry” (CRB “Xenopes”), University of Rennes 1 (http://xenopus.univ-rennes1.fr/). The frogs were fed daily *ad libitum* with pellets of trout food. They were allowed to acclimatize for 3 weeks at 25°C with a photoperiod of 12 hrs:12 hrs in constantly filtered water prior to starting the experiments. All experiments were performed in an animal house accredited by the French Ministry of Animal Welfare (n° B 38 421 10 001) and in accordance with EU laws and the recommendations of the ethics committee (ComEth Grenoble - C2EA - 12).

### *Xenopus*exposure to benzo[a]pyrene

*Xenopus* were exposed individually at 25°C in 1 L glass jars protected from the light (3 individual replicates by time and exposure conditions). The exposure solutions consisted of 500 mL tap water (pH 7.8, organic carbon = 0.6 mg.L^-1^, dissolved oxygen = 9.9 mg.L^-1^, nitrate = 2.9 mg.L^-1^, nitrites < 0.02 mg.L^-1^) containing BaP at an initial concentration of 10 μg.L^-1^. This concentration was chosen to match BaP concentrations usually found in highly- polluted waters, as used in previous experiments [16, 65]. The control frogs were exposed to ethanol (vehicle) at 1/1000 (v:v) concentration. To avoid any circadian effects, experiments with the control and BaP-exposed animals were conducted in parallel. The frogs were not fed during the exposure period (from 0 to 24 hours).

### BaP extraction from water and HPLC analysis

Water samples were stored in 50 mL tubes at -20°C. When measuring the BaP concentration, the water samples were defrosted overnight at room temperature. Fifty microliters of fluoranthene (50 mg.L^-1^, extraction internal control) and 5 mL of acetone were added. The solutions were then extracted three times with 30 mL *n*-hexane. The pooled *n*-hexane solution was dehydrated by adding 2.5 g of sodium salt of sulfurous acid (Na_2_SO_3_). This solution was filtered in a glass filter. The remaining salt was agitated twice with 15 mL of *n*-hexane (5 min) and filtered. The filtered *n*-hexane solution was evaporated at 40°C using a rotary evaporator until a volume of 1 mL was obtained. Samples and washing solutions were transferred into a HPLC-brown glass vial and evaporated at room temperature overnight. Afterwards, 500 μL of acetonitrile was put into the vial and was vortexed and sonicated (2 min) before HPLC analysis.

HPLC measurements were carried out on a 1260 infinity LC system (Agilent) equipped with a C18 column (Agilent Zorbax Eclipse PAH). The injection volume was 10 μL. The mobile phase consisted of a gradient of acetonitrile in H_2_O with 0.1% of trifluoroacetic acid (TFA water) from 60% to 100% of acetonitrile for 20 min at a flow rate of 1 mL.min^-1^. PAHs were detected using a fluorescent detector (excitation wavelength of 260 nm and emission wavelength of 420 nm). The following retention times were determined for BaP (14 min) and the internal standard fluoranthene (5 min). A calibration curve of BaP (0, 1, 2.5, 5, 10 mg.L^-1^) was used to determine the sample BaP concentration. The extraction method is 95% efficient for BaP and 97% for fluoranthene. A BaP solution (10 μg.L^-1^) without *Xenopus* was processed as previously described in order to validate the stability of BaP during the 24-hour time period in our conditions (extraction efficiency 97%).

### Sample preparations

The *Xenopus* were sacrificed rapidly at 6, 12, 18 and 24 hours post-exposure with a blow to the head. The liver and gall bladder were rapidly removed. Blood was sampled by means of an intra-cardiac puncture in order to measure glucose levels. All the subsequent experiments (dissection, histology, mRNA sequencing etc.) were performed on the same individuals. For the mRNA sequencing experiments, the same liver lobe from each frog was stored in RNAlater (Ambion, Austin, TX, USA) at 4°C until RNA extraction, while the rest of the organ was embedded in Tissue OCT (Labonord, France), immediately dropped into liquid nitrogen, and conserved at -80°C for further histological analysis. The gall bladders were put into clean tubes, immediately dropped into liquid nitrogen, and conserved at -80°C for BaP metabolism analysis.

### RNA extraction and double-strand cDNA library preparation for sequencing

For each biological replicate, total RNA was extracted from 15 mg liver using the RNAqueous®-4PCR Kit (Ambion, USA) according to the manufacturer's instructions. Total RNA quality and quantity were controlled on an Agilent 2100 Bioanalyzer (Agilent, USA). After extraction, total RNA from each biological replicate was pooled equally in order to obtain 8 μg of total RNA in 50 μL for each treatment and used for mRNAseq library preparation using mRNA-Seq-8 Sample Prep Kit (Illumina, USA) according to the manufacturer's instructions. The mRNAs from each treatment were purified using poly-T beads and chemically fragmented. These fragmented mRNAs were used to prepare double-strand fragmented cDNA using superscript II (Invitrogen) at 42°C for 50 min for the first strand. Second-strand cDNAs were then synthesized and the mRNAs removed using DNA pol I and RNase H at 16°C for 2.5 hours. Double-strand cDNAs were purified using a QIAquick PCR Purification Kit (QIAGEN, Germany) and processed for end repair and 3’ adenylation using Klenow polymerase. These adenylated double-stranded DNA were purified using a MinElute PCR Purification Kit (QIAGEN, Germany) before ligating paired-end adaptors. Products of ligation were purified on 2% agarose gel based on size range (200 ± 25 pb) for downstream enrichment. The adapter-ligated cDNA library was then enriched using PCR with two primers (primer PE 1.0 and primer PE 2.0, sequences of primers are available at http://illumina.com.) annealing to the end of adapters and Phusion DNA polymerase (Finnzymes Oy). PCR cycles were 30 s at 98°C followed by 15 cycles of 10 s at 98°C, 30 s at 65°C, 30 s at 72°C and a final elongation step of 5 min at 72°C. The enriched cDNA libraries were then purified using a QIAquick PCR Purification Kit (QIAGEN, Germany) before quality control analysis on an Agilent 2100 Bioanalyzer.

### Sequencing of cDNA libraries, mapping reads on the *Xenopus*genome

Each cDNA library was sequenced on a separated flow cell lane on a Genome Analyzer II (Illumina Corporation) at the National Sequencing Center (Genoscope, Evry, France) to obtain 75 bp single-end reads. Quality control of the reads was performed using the R/Bioconductor package Short-Read v.1.8.2 (http://www.bioconductor.org/packages/2.7/bioc/html/ShortRead.html). The reads were trimmed of adaptor sequences and low-quality 3’ ends using functions from the HTSeq python package (v.0.4.7p2, http://www-huber.embl.de/users/anders/HTSeq), and removed completely when their length was less than 35 bp. The cleaned reads were mapped to the *Xenopus* genome assembly (JGI4.2) with Ensembl annotations (release 62) using Bowtie-0.12.7 / Tophat 1.2 software [66] (http://ccb.jhu.edu/software/tophat/index.shtml) (with a maximum intron size of 250000 bp for the discovery of novel junctions between exons).

### Gene expression quantification and differential analysis

The HTSeq-count function (http://www-huber.embl.de/users/anders/HTSeq/doc/count.html) from the HTSeq package was applied with the option mode = intersection_nonempty to Tophat alignments to enumerate, for each library, the number of short reads overlapping the Ensembl annotated genes. A table containing the counts for each gene in each library was assembled and filtered prior to the next step to ensure only genes represented by at least 20 reads across all libraries were kept. The Bioconductor package DESeq 1.2.1 [67] was then used to normalize short read counts across all libraries and to test for the differential expression of the annotated transcripts between exposed and unexposed animals at each exposure time point. Genes were considered to be differentially transcribed when the transcription ratio TR (BaP-treated versus control) at any time point was >1.5 in either direction with an adjusted P-value (FDR) lower than 0.05 after the multiple testing correction.

### Analysis of gene functions differentially regulated by BaP treatment

A comparative analysis of differentially regulated gene functions in BaP-treated and control animals was performed on the genes showing a significant differential transcription at at least one time point. We manually assigned all detected transcripts to different biological categories based on their Ensembl annotation. Genes were classified into 10 categories: cell death/proliferation, cytoskeleton and extracellular matrix, detoxification enzymes, DNA interaction and repair, carbohydrate metabolism and ATP production, lipid metabolism, protein metabolism, immunity, signaling and transport, and hypothetical proteins. In order to compare the transcription patterns of genes from each category, hierarchical clustering was performed by loading fold transcription values into TM4 Multi experiment Viewer (MeV) software [68]. Gene trees were calculated using Pearson uncentered distance metric and complete linkage method with optimization of gene order [69].

### Functional annotation enrichment

Differentially expressed genes were subjected to annotation enrichment analysis and Kyoto Encyclopedia of Genes and Genomes (KEGG) pathway mapping using the online functional annotation tool DAVID (Database for Annotation, Visualisation and Integrated Discovery, http://david.abcc.ncifcrf.gov/) [70, 71] with all genes detected in our experiment as background (10,483 genes). *Xenopus* gene identifiers were transformed into their human orthologs to improve the richness of the output [72, 73]. In order to differentiate functional enrichment due to up- and down- regulated genes, the DAVID tool was used on any gene found up- or down-regulated at at least one time point from the genes found to be differentially transcribed at any time point. Significance was calculated using a modified Fisher’s Exact test (p < 0.05). Heat maps of expression profiles for genes sharing enriched annotation pathways or other genes associated with these pathways were produced using TM4 Multi experiment Viewer (MeV) software, as described above.

### mRNAseq data validation using RT-qPCR

The transcription levels of ten genes (ENSXETG00000021546 - *Cytochrome P450 26B1*; ENSXETG00000003432 - *Cytochrome P450 51A1*; ENSXETG00000021140 - *Cytochrome P450 1A1*; ENSXETG00000011124 - *jun* oncogene; ENSXETG00000023050 - *squalene epoxidase*; ENSXETG00000007561 – *catalase*; ENSXETG00000011266 *- phosphoenolpyruvate carboxykinase 1; ENSXETG00000024078 - heat* shock 70 kDa protein; ENSXETG00000018041 - *potassium large conductance calcium-activated channel M alpha1*; ENSXETG00000017419 - *pyruvate dehydrogenase phosphatase catalytic subunit 2*) found to be over-transcribed at at least one time point were validated using real-time quantitative PCR (RT-qPCR). We treated 1.6 μg of the total RNA per *Xenopus* and per time point with DNAse I (Invitrogen, Carlsbad, CA, USA) and used cDNA synthesis with superscript III and OligodT20 primer (Invitrogen) according to the manufacturer’s instructions, the resulting cDNAs were diluted 50 times. Real-time quantitative PCR reactions of 25 μL were performed on an iQ5 system (Biorad, Hercules, CA, USA) using iQ SYBR Green supermix (Biorad), 0.3 mM of each primer (Additional file 8: Table S3) and 5 μL of diluted cDNAs. A melting curve analysis was performed to check for the specificity of the primers in producing one single targeted PCR product. The transcription level was quantified using the ΔΔCt method taking into account PCR efficiency [74] and using three housekeeping genes for normalization: the *ribosomal proteins L8* (ENSXETG00000015483), *S7* (ENSXETG00000001209) and *L27* (ENSXETG00000003912). Results were expressed as mean transcription ratio (±SE) between BaP-treated animals and controls for each time point.

### Measurements of blood glucose

Blood glucose concentrations were measured using Accu-Chek® performa teststrips (Roche).

### Cryosectioning

Frozen samples were cut into 7 μm thick sections on a cryostat (CM3050 S, Leica, Nussloch, Germany). Frozen sections were collected on Superfrost plus microscope slide (Labonord). Cryochamber and specimen temperatures were set at -25°C.

### Terminal deoxynucleotidyl transferase dUTP nick end labeling (TUNEL) assay

Hepatic cell apoptosis was examined using terminal deoxynucleotidyl transferase dUTP nick end labeling (TUNEL) assay on frozen sections according to the manufacturer’s instructions (in situ cell death detection kit AP, Roche). After labeling, frozen sections were counterstained with Mayer’s Haemalaun (Merck Millipore) and mounted in aqueous mounting agent (Aquatex, Merck Millipore). The apoptotic positive cells were counted on the basis of 5 high power fields by slide (1,000 to 1,700 counted cells).

### Hematoxylin, eosin and saffron (HES) staining

The amount of pigment in the liver tissue was assessed after section staining according to the standard hematoxylin, eosin and saffron protocol: frozen sections were air dried to the slides, stained with Mayer’s Haemalaun solution (Merck Millipore), with alcoholic eosin Y (1%) and with alcoholic saffron (1%) and mounted in Coverquick 4000 (Labonord) mounting media. In order to quantify pigment content in the HES sections, 24 slides per treatment and time point were photographed under a Nikon Eclipse E600 microscope using an Olympus DP70 digital camera. Between 3 and 5 pictures were randomly taken along the slide, giving a total of 72 RBG pictures. Each picture has been imported into Ecognition Developer [version 8.7, Trimble Geospatial Imaging, Westminster, CO, USA]. We processed chessboard segmentation (1 pixel = 1 object) over the images. Then, using a 2-class typology (pigment or not), we manually defined a brightness threshold to discriminate between the two classes and classified the images according to this threshold. We rasterized the results of the classifications and estimated the proportion of each class.

### Oil red O (ORO) staining

Frozen sections were stained with Oil red O to assess total lipid content according to the standard method: frozen sections were air dried to the slides, fixed for 20 minutes in 4% formaldehyde, rinsed with 60% isopropanol, stained with freshly-prepared Oil Red O (Sigma-Aldrich), rinsed with 60% isopropanol, counterstained with Mayer’s Haemalaun (Merck Millipore) and mounted in aqueous mounting agent (Aquatex, Merck Millipore). In order to quantify lipid content, 21 slides per treatment and time point were photographed using the same system described above. Between 3 and 5 pictures were taken randomly along the slides, giving a total of 69 images. We processed image segmentation using R, G and B layer similarity and leaving the default sharpness and compactness parameters. The size object parameter was set to 10. Then, using a 2-class typology (lipid or not), we manually selected several training parcels for each class. We then ran an image classification using the nearest neighboring algorithm. We rasterized the results of the classifications and estimated the proportion of each class.

### Biliary cholesterol content

The gallbladders were weighed and thawed at room temperature and extracted twice with a 45% ethanol mixture. They were centrifuged for 5 min at 10,000 rpm and 100 μL of supernatant was collected. 5α-cholestane (Sigma-Aldrich) and 500 μL of a KOH 5 M solution in methanol were added as an internal standard and as a saponification reagent, respectively. After a one hour reaction at 55°C, 500 μL of water was added and NaCl was poured until saturation. Three mL of *n*-hexane were added and the mixture centrifuged for 5 min at 3,000 rpm. The *n*-hexane phase was removed and stored. Then 3 mL of diethylether was added for the second extraction. The samples were centrifuged for 5 min at 3,000 rpm and the diethylether phase was removed and combined to the *n*-hexane phase. *n*-hexane and diethylether were evaporated with nitrogen, and the samples were derivatized for 1 hour at 55°C with 50 μL of 1%TMCS in BSTFA (Sigma-Aldrich) before being subjected to GC-MS analysis (Varian 320). The GC-MS parameters were as follows: injection temperature was 300°C in splitless mode; separation was performed on a DB-5MS column (Agilent) 30 m x 0.25 mm x 0.25 μm with Heat 1 mL.min^-1^ as carrier gas and a temperature gradient from 240°C to 300°C at 20°C.min^-1^; ion source and transfer line were heated at 250°C; cholesterol and 5α-cholestane were quantified in SIM mode using the following ions, 329 m/z and 217 m/z, respectively; confirmation ions for cholesterol were 458 m/z and 368 m/z, and 372 m/z and 357 m/z for 5α-cholestane; The quantification limit for cholesterol was 0.2 μg.mL^-1^ of bile.

### Biliary BaP metabolite content

Total biliary metabolite content was determined by fluorescence [75]. Forty-five percent ethanolic extracts obtained as described above, were diluted 10 and 100 times in 45% ethanol before fluorescence intensity measurement at λ_ex/em_ = 380/430 nm [76] using Varioscan flash (Thermoscientific). The quantity of BaP metabolites produced was expressed in μg.L^-1^ equivalent 3-hydroxy-benzo[a]pyrene (3-OHBaP, NCI Chemical Carcinogen Standard Repository) used herein as standard for quantification.

### Statistical analysis

Data are expressed as the mean ± SEM. Each value was derived from three individual experiments. Since the nature of the distribution of the results was unknown (Gaussian or not), the statistical significance between the means obtained for BaP-exposed *Xenopus* and the controls were assessed using the non-parametric Mann and Whitney’s U test of significance (p < 0.05 was considered statistically significant).

### Data access

Detailed transcription data for all genes detected by RNAseq in the present study are presented in Additional file 2: Table S1. RNA-seq sequence data are available in the ArrayExpress database (http://www.ebi.ac.uk/arrayexpress) under accession number E-MTAB-2444.

## Electronic supplementary material

Additional file 1: Figure S1: Sequencing and mapping statistics and differential transcription analysis. A. Sequencing and mapping statistics. B. Differential transcription analysis. C. Venn diagram showing the number of genes differentially transcribed at any given time point compared to the control. The total number of genes differentially transcribed at each time point is indicated in brackets. (PPTX 810 KB)

Additional file 2: Table S1: List of all genes detected in mRNAseq. For each gene, *Xenopus* Ensembl ID and their human orthologs Ensembl ID are indicated together with their transcription level (fold) compared to the control at each time point, and with adjusted p value. (XLSX 1 MB)

Additional file 3: Table S2: List of the genes significantly differentially transcribed after BaP exposure. For each gene, *Xenopus* Ensembl ID and their human orthologs Ensembl ID are indicated together with their transcription level (fold) compared to the control at each time point, and with adjusted p value. (XLSX 59 KB)

Additional file 4: Figure S2: Quantitative real-time PCR validation of mRNAseq data. The correlation between mRNAseq and RT-qPCR data was performed on transcription ratios obtained at each time point for 10 transcripts showing a significant differential transcription in at least one time point of exposure. The blue dashed line represents an equal transcription ratio between both techniques. (PPTX 99 KB)

Additional file 5: Figure S3: Map overview of significant changes in liver gene transcription in *Xenopus tropicalis* in response to BaP exposure. Genes have been manually assigned to general biological pathways. Color scale indicates transcription ratios relative to the control. (PPTX 3 MB)

Additional file 6: Figure S4: Hierarchical clustering of genes involved in proliferation/apoptosis processes found differentially transcribed compared to the control. A. Hierarchical clustering of genes involved in apoptosis processes. B. Hierarchical clustering of genes involved in proliferation processes. Color scale indicates transcription ratios relative to the control. Gene names or annotations are indicated. Stars indicate significant transcription variations (>1.5-fold in either direction and corrected p < 0.05). (PPTX 3 MB)

Additional file 7: Figure S5: Cell-cell adhesion disturbance induced by BaP. A. Hierarchical clustering of tight and adherent junction genes found differentially transcribed compared to control. Color scale indicates transcription ratios relative to the control. Gene names are indicated. Stars indicate significant transcription variations (>1.5-fold in either direction and corrected p < 0.05). B. Hematoxylin-eosine-safran (HES) staining of liver sections from control and *X. tropicalis* exposed to BaP showing histopathological changes in cell-cell contact in BaP-treated livers compared to control. (a) Sections shown in low magnification (100×). (b) High magnification (400x) of areas delimited by dashed line. H, hepatocyte; m, membrane; n, nucleus; v, vessel. (PPTX 12 MB)

Additional file 8: Table S3: Primer sequences used for RT-qPCR in mRNAseq data validation. (DOC 55 KB)
