# Supplementary figures and images for: Impaired liver function in Xenopus tropicalis exposed to benzo[a]pyrene: transcriptomic and metabolic evidence
Source: BMC Genomics. 2014 Aug 8;15(1):666. doi: 10.1186/1471-2164-15-666 (PMC4141109; doi:10.1186/1471-2164-15-666)

## Slide 1
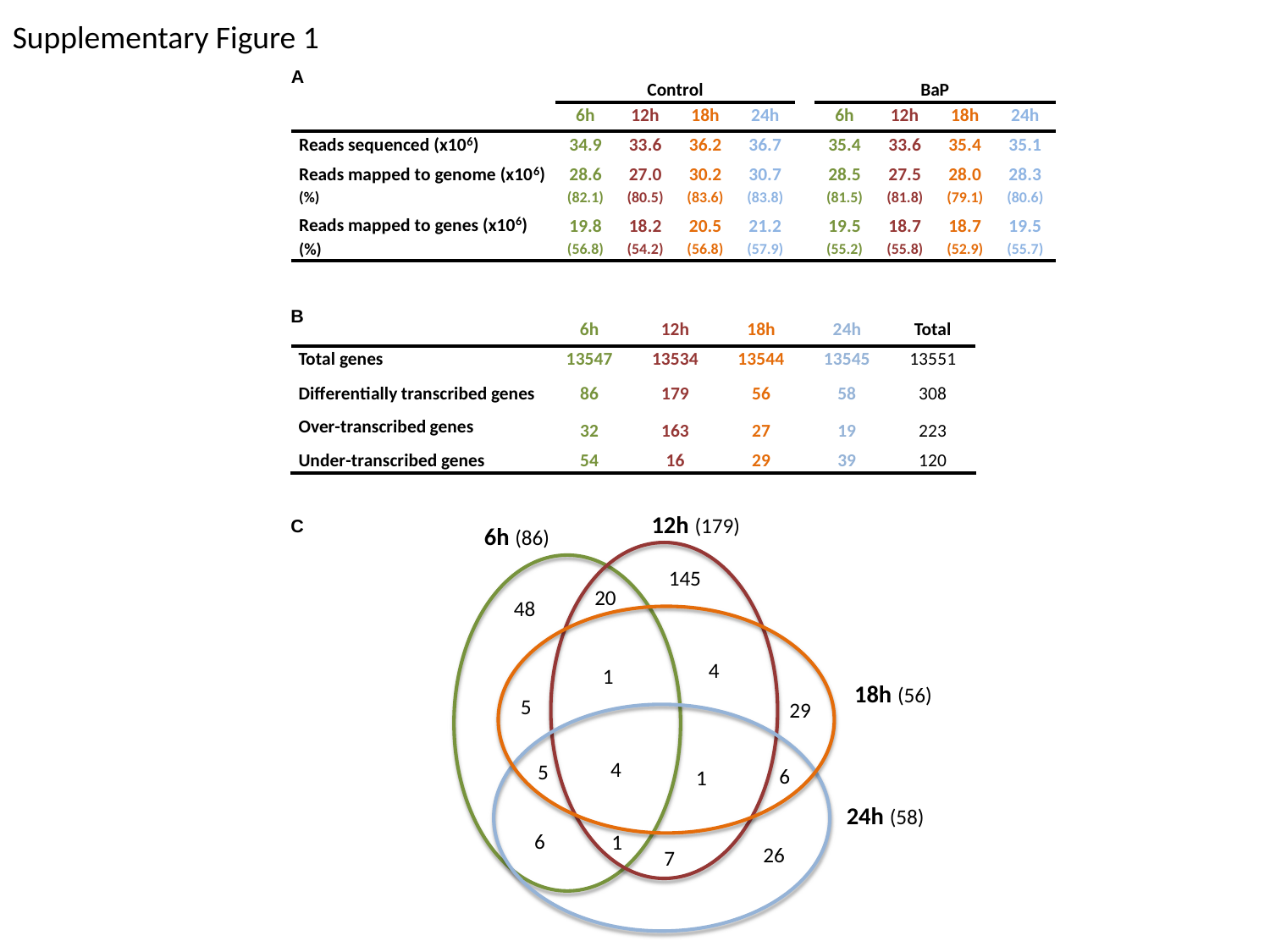

Supplementary Figure 1

Supplement: Supplementary file 1 — Additional file 1: Figure S1: Sequencing and mapping statistics and differential transcription analysis. A. Sequencing and mapping statistics. B. Differential transcription analysis. C. Venn diagram showing the number of genes differentially transcribed at any given time point compared to the control. The total number of genes differentially transcribed at each time point is indicated in brackets. (PPTX 810 KB) [file 12864_2014_6364_MOESM1_ESM.pptx]

## Slide 1
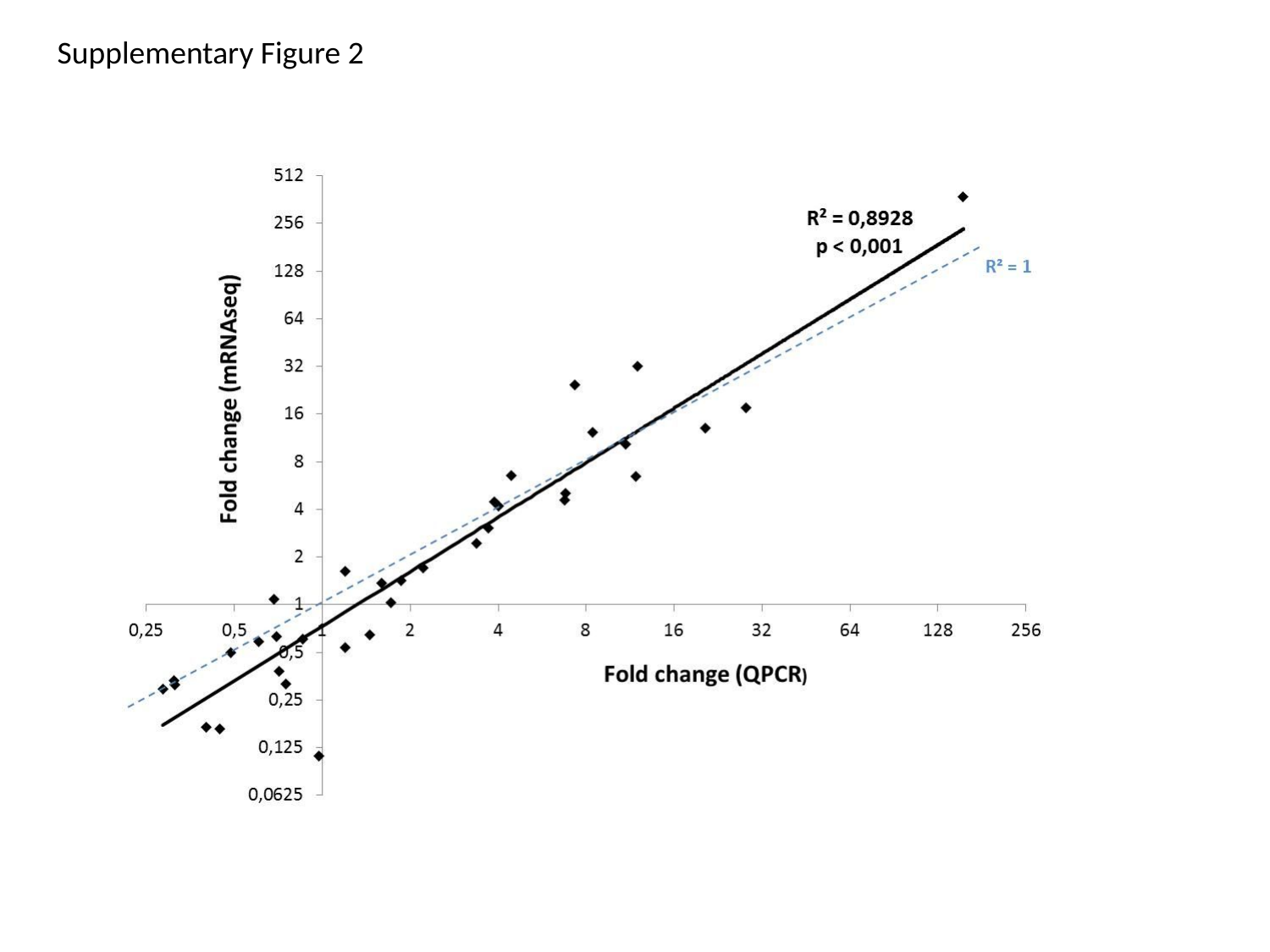

Supplementary Figure 2

Supplement: Supplementary file 4 — Additional file 4: Figure S2: Quantitative real-time PCR validation of mRNAseq data. The correlation between mRNAseq and RT-qPCR data was performed on transcription ratios obtained at each time point for 10 transcripts showing a significant differential transcription in at least one time point of exposure. The blue dashed line represents an equal transcription ratio between both techniques. (PPTX 99 KB) [file 12864_2014_6364_MOESM4_ESM.pptx]

## Slide 1
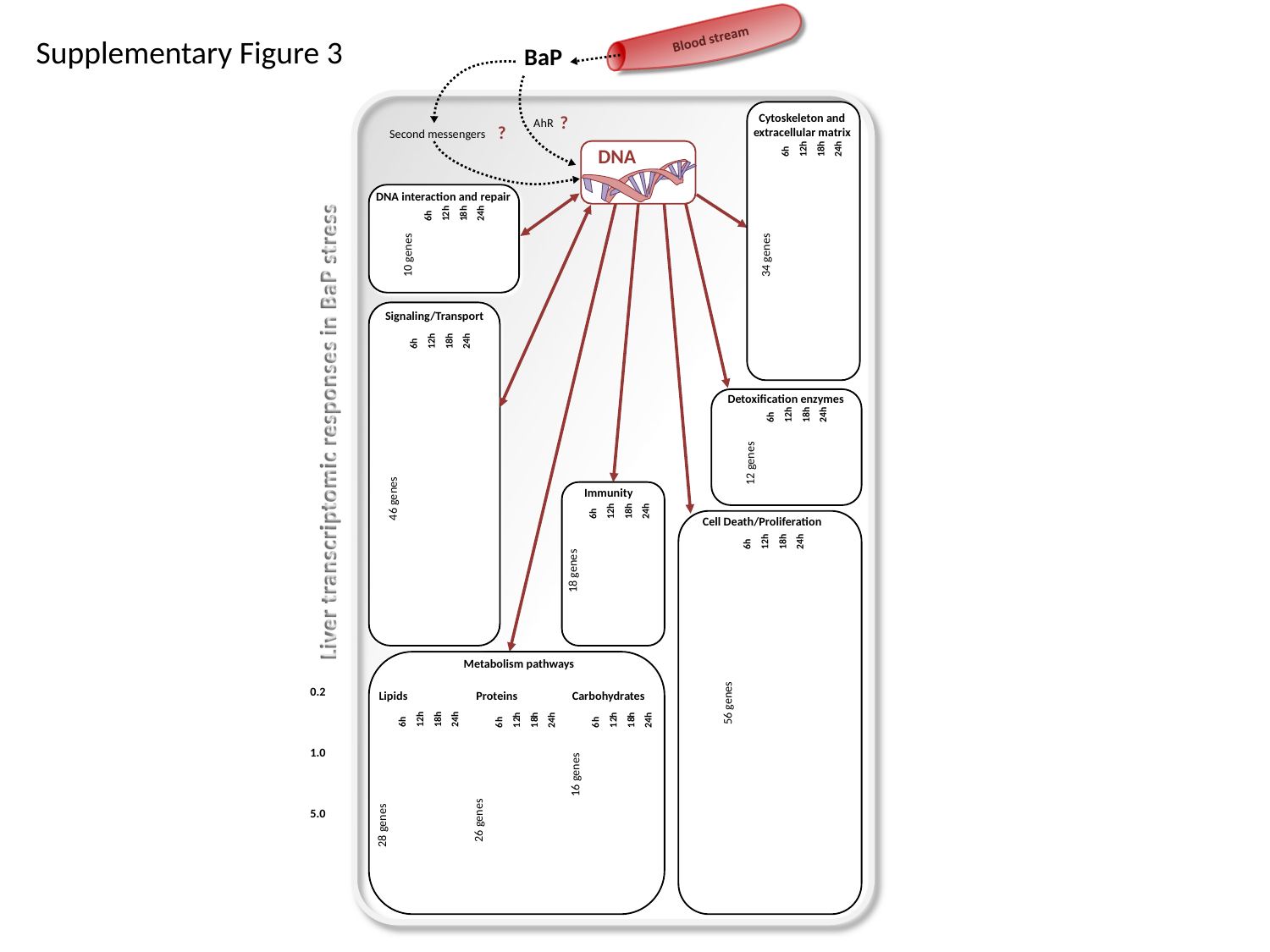

Supplementary Figure 3

Supplement: Supplementary file 5 — Additional file 5: Figure S3: Map overview of significant changes in liver gene transcription in Xenopus tropicalis in response to BaP exposure. Genes have been manually assigned to general biological pathways. Color scale indicates transcription ratios relative to the control. (PPTX 3 MB) [file 12864_2014_6364_MOESM5_ESM.pptx]
