# Supplementary material for: Impaired liver function in Xenopus tropicalis exposed to benzo[a]pyrene: transcriptomic and metabolic evidence
Source: BMC Genomics. 2014 Aug 8;15(1):666. doi: 10.1186/1471-2164-15-666 (PMC4141109; doi:10.1186/1471-2164-15-666)
Supplement: Supplementary file 6 — Additional file 6: Figure S4: Hierarchical clustering of genes involved in proliferation/apoptosis processes found differentially transcribed compared to the control. A. Hierarchical clustering of genes involved in apoptosis processes. B. Hierarchical clustering of genes involved in proliferation processes. Color scale indicates transcription ratios relative to the control. Gene names or annotations are indicated. Stars indicate significant transcription variations (>1.5-fold in either direction and corrected p < 0.05). (PPTX 3 MB) [file 12864_2014_6364_MOESM6_ESM.pptx]

## Slide 1
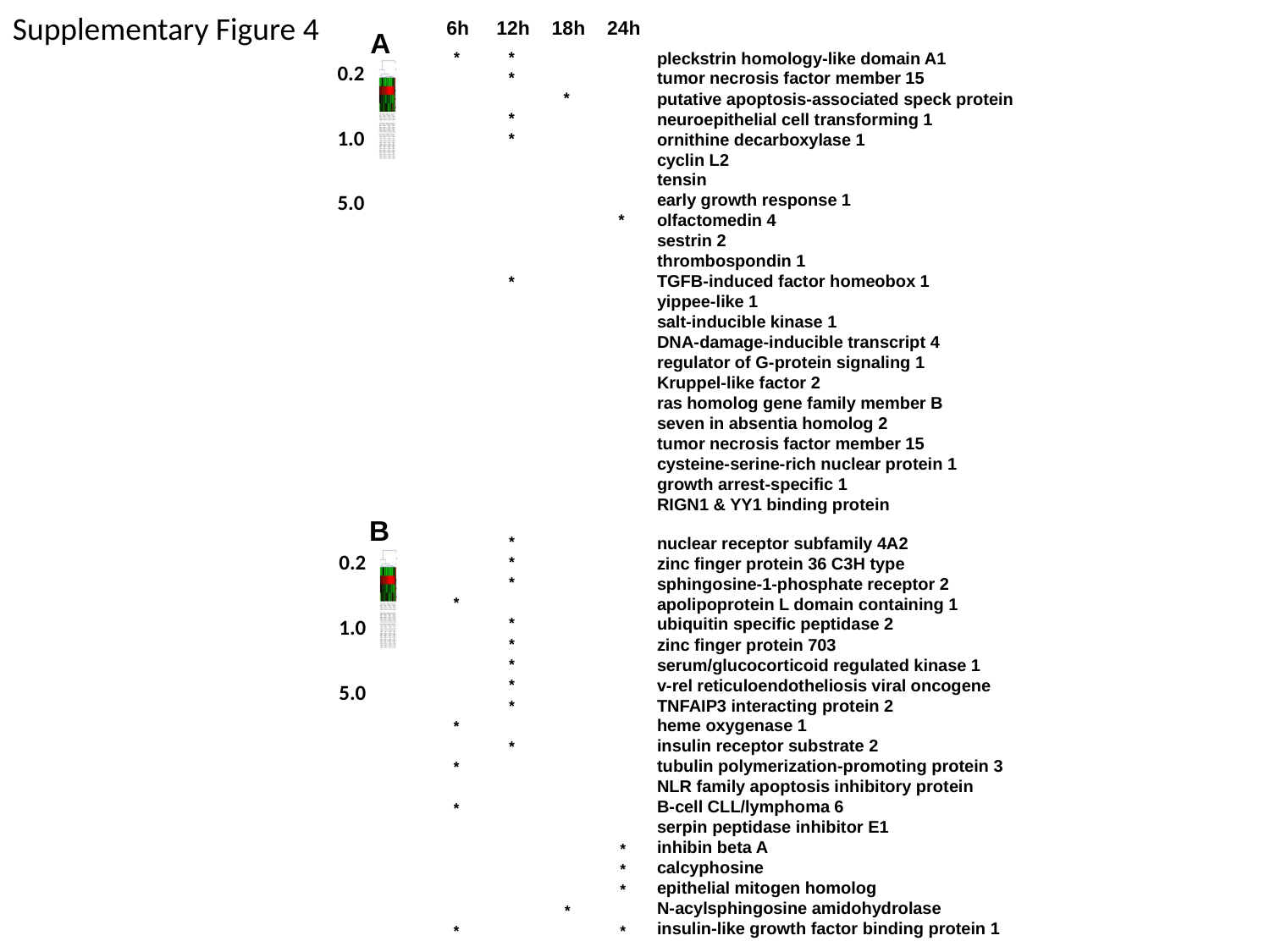

Supplementary Figure 4
 6h 12h 18h 24h
 A
pleckstrin homology-like domain A1
tumor necrosis factor member 15
putative apoptosis-associated speck protein
neuroepithelial cell transforming 1
ornithine decarboxylase 1
cyclin L2
tensin
early growth response 1
olfactomedin 4
sestrin 2
thrombospondin 1
TGFB-induced factor homeobox 1
yippee-like 1
salt-inducible kinase 1
DNA-damage-inducible transcript 4
regulator of G-protein signaling 1
Kruppel-like factor 2
ras homolog gene family member B
seven in absentia homolog 2
tumor necrosis factor member 15
cysteine-serine-rich nuclear protein 1
growth arrest-specific 1
RIGN1 & YY1 binding protein
0.2
1.0
5.0
 B
nuclear receptor subfamily 4A2
zinc finger protein 36 C3H type
sphingosine-1-phosphate receptor 2
apolipoprotein L domain containing 1
ubiquitin specific peptidase 2
zinc finger protein 703
serum/glucocorticoid regulated kinase 1
v-rel reticuloendotheliosis viral oncogene
TNFAIP3 interacting protein 2
heme oxygenase 1
insulin receptor substrate 2
tubulin polymerization-promoting protein 3
NLR family apoptosis inhibitory protein
B-cell CLL/lymphoma 6
serpin peptidase inhibitor E1
inhibin beta A
calcyphosine
epithelial mitogen homolog
N-acylsphingosine amidohydrolase
insulin-like growth factor binding protein 1
0.2
1.0
5.0
