# Supplementary material for: Impaired liver function in Xenopus tropicalis exposed to benzo[a]pyrene: transcriptomic and metabolic evidence
Source: BMC Genomics. 2014 Aug 8;15(1):666. doi: 10.1186/1471-2164-15-666 (PMC4141109; doi:10.1186/1471-2164-15-666)
Supplement: Supplementary file 7 — Additional file 7: Figure S5: Cell-cell adhesion disturbance induced by BaP. A. Hierarchical clustering of tight and adherent junction genes found differentially transcribed compared to control. Color scale indicates transcription ratios relative to the control. Gene names are indicated. Stars indicate significant transcription variations (>1.5-fold in either direction and corrected p < 0.05). B. Hematoxylin-eosine-safran (HES) staining of liver sections from control and X. tropicalis exposed to BaP showing histopathological changes in cell-cell contact in BaP-treated livers compared to control. (a) Sections shown in low magnification (100×). (b) High magnification (400x) of areas delimited by dashed line. H, hepatocyte; m, membrane; n, nucleus; v, vessel. (PPTX 12 MB) [file 12864_2014_6364_MOESM7_ESM.pptx]

## Slide 1
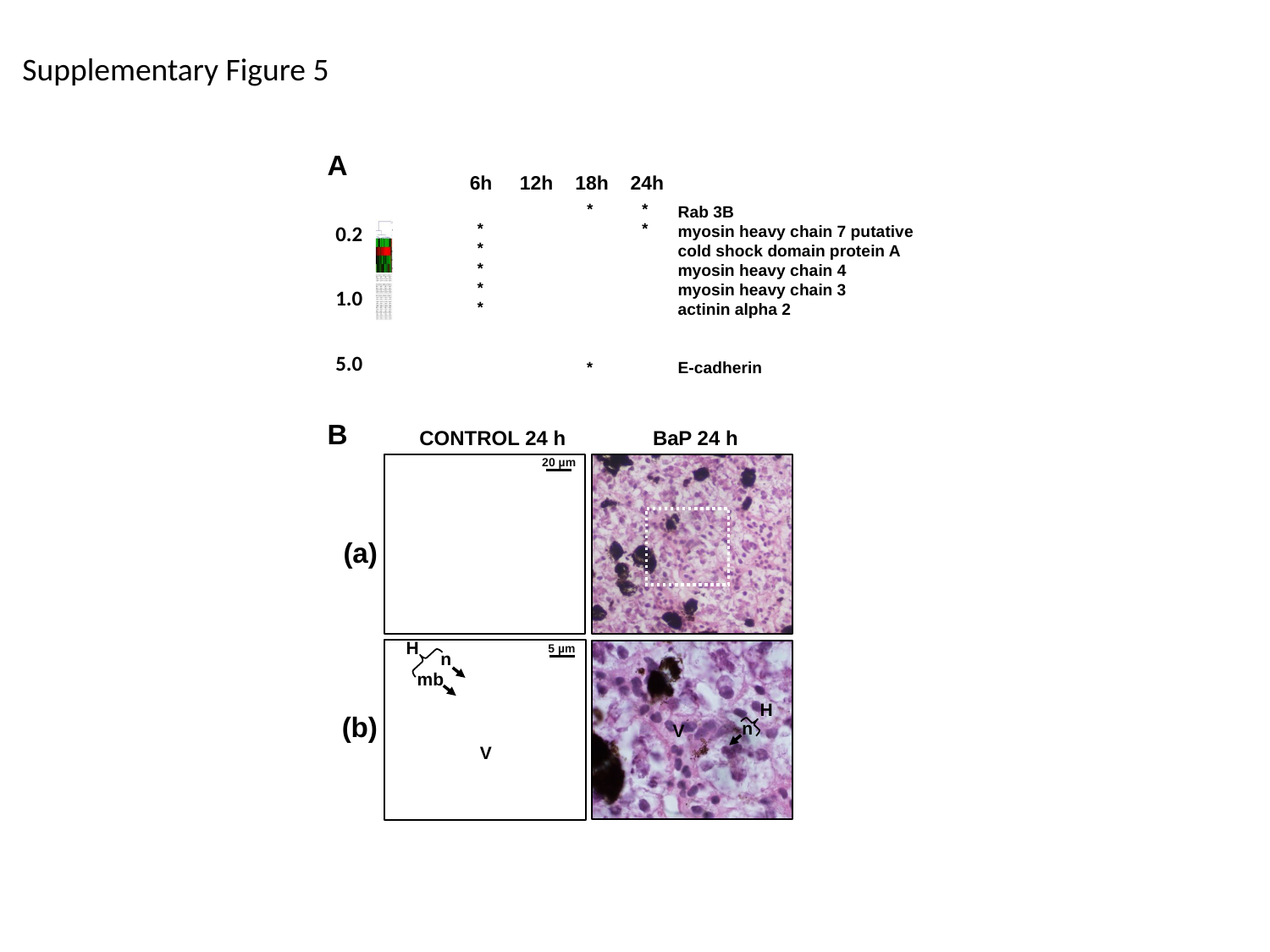

Supplementary Figure 5
 A
 6h 12h 18h 24h
Rab 3B
myosin heavy chain 7 putative
cold shock domain protein A
myosin heavy chain 4
myosin heavy chain 3
actinin alpha 2
E-cadherin
0.2
1.0
5.0
 B
CONTROL 24 h
BaP 24 h
 (a)
 (b)
