# Supplementary material for: Impaired liver function in Xenopus tropicalis exposed to benzo[a]pyrene: transcriptomic and metabolic evidence
Source: BMC Genomics. 2014 Aug 8;15(1):666. doi: 10.1186/1471-2164-15-666 (PMC4141109; doi:10.1186/1471-2164-15-666)
Supplement: Supplementary file 8 — Additional file 8: Table S3: Primer sequences used for RT-qPCR in mRNAseq data validation. (DOC 55 KB) [file 12864_2014_6364_MOESM8_ESM.doc]

|  | | | | |
| --- | --- | --- | --- | --- |
| Gene name/annotation | Accession no. | Forward primer | Reverse primer | Annealing T°C |
|  |  |  |  |  |
| cytochrome P450 CYP26B1 | ENSXETG00000021546 | TTCTACGCCTGCCTTATCTTAC | CTGGGTTTGGATGGTCTACG | 58 |
| cytochrome P450 CYP51A1 | ENSXETG00000003432 | AGGTCGGCATCGCTGTATC | ATGGTTGTGTAGTTTATCGTAGGG | 55 |
| cytochrome P450 CYP1A1 | ENSXETG00000021140 | GATACCATTACCACTGCTCTCTC | CCTCCTTTCTCTGCCAATAACC | 53 |
| jun oncogene | ENSXETG00000011124 | TCACAGACGAGCAAGAAG | TCACAGACGAGCAAGAAG | 51 |
| squalene epoxidase | ENSXETG00000023050 | TTGCGGTAGAGAACGACAG | CATTCAGCACTACACTCATCC | 52 |
| catalase | ENSXETG00000007561 | AGTGCCCAAAGAAGGAATC | GGTTAATGAAGCGGAGAGG | 58 |
| phosphoenolpyruvate carboxykinase 1 | ENSXETG00000011266 | ATGCCGACCTGCCGTATG | TCCCACCCTCACTCACAAC | 60 |
| heat shock 70kDa protein | ENSXETG00000024078 | ACTTACTTCTGCTGGATGTTG | AGGTCTGTGTCTGCTTGG | 51 |
| K calcium-activated channel M alpha1 | ENSXETG00000018041 | GGATGGTGGCTGTTATGGAGAC | TGGCTGGGTGTGCTGAGG | 56 |
| pyruvate dehydrogenase phosphatase | ENSXETG00000017419 | TCGCCTTCAAACTACCATAC | GACCACTTGCTCATTCTCC | 59 |
| ribosomal proteins L8 | ENSXETG00000015483 | GCCACCGTTATCTCCCACAATC | GACCACCACCAGCAACAACC | 56 |
| ribosomal proteins S7 | ENSXETG00000001209 | GGTATTTCCCAGGCTCTCTTG | ACCACCTCCAACTTCTATTTCC | 54 |
| ribosomal proteins L27 | ENSXETG00000003912 | GTTGTCCTTGTCCTAGCAG | TGGCAGTCACCTTACGAG | 58 |
|  |  |  |  |  |
